# OVERVIEW OF COVID-19 CASES IN PREGNANCY AT THE HOSPITAL UNIVERSITAS SUMATERA UTARA, INDONESIA, WHEN THE PANDEMIC STORM HIT IN THE 2020-2022 PERIOD.

**DOI:** 10.21010/Ajidv17i2S.4

**Published:** 2023-08-01

**Authors:** Muhammad Ernesto Azguevara Ganis Siregar, Henry Salim Siregar, Muara Panusunan Lubis, Ichwanul Adenin, Irwin Lamtota Lumbanraja

**Affiliations:** 1 Medical student, Faculty of Medicine, Universitas Sumatera Utara, Medan, Indonesia; 2 Department of Obstetrics and Gynecology, Faculty of Medicine, Universitas Sumatera Utara, Medan, Indonesia

**Keywords:** Coronavirus 2019, fetomaternal outcomes, pregnancy, Severe Acute Respiratory Syndrome-Coronavirus-2

## Abstract

**Background:**

COVID-19 (Coronavirus 2019) is caused by SARS-CoV-2 (Severe Acute Respiratory Syndrome-Coronavirus-2), an acute infectious disease primarily affecting the respiratory system. Data on COVID-19 exposure during pregnancy and issues associated with COVID-19 during pregnancy remain limited. This research aimed to determine the number of pregnant women infected by COVID-19, laboratory test findings of pregnant women related to COVID-19 infection, infant outcome from mother with or without COVID-19 infection and referential status for COVID-19 and non-COVID-19 pregnant women at the USU Hospital during the 2020–2022 period.

**Materials and Methods:**

This research was conducted using a descriptive method with a cross-sectional study approach using a non-probability sampling technique by collecting secondary data from COVID-19 and non-COVID-19 pregnant women at the USU Hospital during the 2020-2022 period, where 112 samples were obtained.

**Results:**

The majority of COVID-19 pregnant women and non-COVID-19 pregnant women have been identified sequentially based on Hb (11.6%-decreased vs 79.5%-normal); Ht (11.6%-decreased vs 76.8%-increased); leukocytes (11.6%-increased vs 83%-normal); thrombocytes (8.9%-normal vs 86.6%-normal); PT (9.8%-normal vs 50.9%-normal); APTT (11.6%-normal vs 87.5%-normal); D-dimer (11.6%-long vs 56.3%-long); procalcitonin (7.1%-increased vs 87.5%-normal); NLR (8%-increased vs 82.1%-normal); CRP (12.5%-increased vs 87.5%-normal) and all of the baby outcomes were non-COVID-19 and the majority of pregnant women were not referred.

**Conclusion:**

Based on the data in this study, the majority of pregnant women and babies at the USU Hospital during the 2020–2022 period were non-COVID-19 positive and with non-referral status. Laboratory findings of COVID-19 in pregnancy significantly reveals abnormalities.

## Introduction

COVID-19 (Coronavirus 2019), caused by SARS-CoV-2 (Severe Acute Respiratory Syndrome-Coronavirus-2), an acute infectious disease primarily affecting the respiratory system, was first detected in Wuhan, China, in December 2019 and then spread across borders due to transmission from human to human through intercontinental travel. (Al‐Jarallah *et al.*, 2021) On March 11, 2020, the WHO (World Health Organization) declared that this condition was a pandemic. Globally, 1,056,186 people have died from COVID-19. Until early October 2020, the number of new cases reported had reached 300,000 per day.( Allotey *et al.*, 2020; Al‐Jarallah *et al.*, 2021; Bonnesen *et al.*, 2021)

One of the countries affected by a severe infection with SARS-CoV-2 is Indonesia. As of October 9, 2020, Indonesia reported 324,658 confirmed cases of COVID-19, of which 247,667 people recovered and 11,677 people died. Until March 3, 2022, the number of cases reached 5,589,176, with a total death toll of 148,660. (Parisa Maleki Dana *et al.*, 2020; Covali *et al.*, 2021)

SARS-CoV-2 is transmitted by respiratory droplets, aerosol and close person to person contact. The virus enters the target cells via the SARS-CoV-2 glycoprotein (S) spike that binds to the host’s ACE-2 (angiotensin-converting enzyme-2) receptor. In addition, host cell proteases, such as the TMPRSS2 (transmembrane biserine protease) then cleave the viral S protein, allowing permanent fusion of the virus and the host cell membrane. (Bonnesen *et al.*, 2021)

Pregnant women were identified as a vulnerable group because they have greater risk of complication and severe disease from infection especially COVID-19 infection. Therefore, pregnant women were advised to take additional precautions from COVID-19. Pregnant women with COVID-19 will undergo numerous changes in response to infection. COVID-19 will affect respiratory function, coagulation, endothelial cell function and also the placenta. Several studies reported the COVID-19 infection during pregnancy also increases the risk of adverse outcomes for the child. Maternal-fetal transmission through haematogenous enters placenta, chorionic villous and fetal blood vessel is known but appears to occur in a small number of cases in COVID-19 infection. (Dumitriu *et al.*, 2021; Lagunas‐Rangel, 2020) However, it is necessary to separate infants from mother immediately to avoid the transmission from other mechanism. Most pregnant women will have mild or moderate symptoms because the virus is cleared effectively by the immune system. If the immune system does not react appropriately and have other risk factor such as older maternal, high body mass index, diabetes and hypertension, pregnant women will experience severe symptoms. (Levi *et al.*, 2020) Mothers with severe symptoms will have problems in both the mother and the infant, this condition need advanced management so it must be referred.(Liu *et al.*, 2020)

March 2022 is two years since COVID-19 emerged as a pandemic in Indonesia. Vaccination, which is currently being promoted to Indonesian citizens is hope to reduce the transmission, illness, and deaths of COVID-19, especially among pregnant women. (Marsden *et al.*, 2021; LUMBANRAJA *et al.*, 2023) Despite the large number of COVID-19 cases and even deaths, data on exposure to COVID-19 during pregnancy and problems related to COVID-19 during pregnancy are still limited. The purpose of this study was to describe cases of COVID-19 in pregnant women who were treated at the USU Hospital during the 2020–2022 period.

## Material and Methods

This study utilised a cross-sectional study design at the USU Hospital from August 2022 to November 2022, with the data collection method in the form of medical record data. The research sample was COVID-19 and non-COVID-19 pregnant patients at the USU Hospital for the 2020–2022 period and the sampling method used was total sampling. The inclusion criteria in this study were pregnant women with COVID-19 and non-COVID-19, while the exclusion criteria were patients with incomplete data. Secondary data consists of patient identity and laboratory test results.

The study was approved by ethics committee of Universitas Sumatera Utara with registration number 668/KEPK/USU/2022.

## Statistical Analytic

An overview of the research subject will be presented in a concise and detailed form. Categorical data is presented in the form of n (%), while numerical data is presented in the form of mean + SD. Data are presented in the form of Tables.

## Results

This study took 112 samples of pregnant women divided into two groups: 98 samples are non-COVID-19 pregnant women and 14 samples are COVID-19 pregnant women. Table 1 shows characteristics of the sample. The highest proportion of this research sample ages in each group was found in the 20-35 years age group. The group of non-COVID-19 pregnant women had the largest proportion compared to the group of pregnant women with COVID-19 (87.5% vs 12.5%). The research subjects had different proportions of parity in each group. The majority of non-COVID-19 pregnant women and COVID-19 pregnant women were multiparous (58.9% and 41.1%, respectively). Based on the region of origin, it showed that the highest proportion of sample hometown is in Medan City (78.6%; 21.4%).

**Table 1:** Sample Characteristics

Variable	Category	Total (n,%)	COVID-19 (n,%)	Non-COVID-19 (n,%)
Age (years	< 20	2 (1,8%)	0 (0,0%)	2 (1,8%)
old)	20-35	88 (78,6%)	8 (7,1%)	80 (71,4%)
	> 35	22 (19,6%)	6 (5,4%)	16 (14,3%)
Parity	Primiparous	46 (41,1%)	1 (0,9%)	45 (40,2%)
	Multiparous	66 (58,9%)	13 (11,6%)	53 (47,3%)
Hometown	Medan City	88 (78,6%)	11 (9,8%)	77 (68,8%)
	Others	24 (21,4%)	3 (2,7%	21 (18,8%)

Table 2 shows laboratory examinations in pregnant women. As shown in Table 2 the majority of COVID-19 pregnant women had lower hemoglobin levels, namely 13 people (11.6%) and non-COVID-19 pregnant women have normal hemoglobin levels, namely 89 people (79.5%). 13 people of COVID-19 pregnant women (11.6%) had decreased haematocrit levels and 86 people of non-COVID- (76.2%) had increased haematocrit levels. The results also showed that the majority of COVID-19 pregnant women had elevated leukocyte levels, namely 13 people (11.6%) and non-COVID-19 pregnant women had normal leukocyte levels, namely 93 people (83.0%). 97 pregnant women with COVID-19 and 10 non-COVID-19 pregnant women had normal platelet levels (8.9% and 86.6%, respectively). Most of the COVID-19 pregnant women had elevated NLR levels, namely 9 people (8.0%) and most of the non-COVID-19 pregnant women had normal NLR levels, namely 92 people (82.1%). 11 pregnant women with COVID-19 and 57 non-COVID-19 pregnant women had normal PT levels (9.8% and 50.9%, respectively). The results also showed that the majority of pregnant women with COVID-19 (98 people) and non-COVID-19 pregnant women (13 people) had normal APTT levels (11.6% and 87.5%, respectively). The majority of pregnant women with COVID-19 (13 people) and non-COVID-19 pregnant women (63 people) had prolonged D-Dimer levels (11.6% and 56.3%, respectively). The results also showed that the majority of COVID-19 pregnant women had elevated procalcitonin levels, namely 8 people (7.9%), and non-COVID-19 pregnant women had normal procalcitonin levels, namely 98 people (87.5%). The results also showed that the majority of COVID-19 pregnant women had elevated CRP levels, namely 14 people (12.5%), and non-COVID-19 pregnant women had normal CRP levels, namely 98 people (87.5%). The majority of pregnant women with COVID-19 (12 people) and non-COVID-19 pregnant women (91 people) had normal fibrinogen levels (10.7% and 81.3%, respectively).

**Table 2:** Laboratory Examination of Pregnant Women

Variables	Category	Total (n,%)	COVID-19 (n,%)	Non-COVID-19 (n,%)
Haemoglobin	Anemia	22 (19,6%)	13 (11,6%)	9 (8,0%)
	Normal	90 (80,4%)	1 (0,9%)	89 (79,5%)
Hematocrite	Decreased	20 (17,9%)	13 (11,6%)	7 (6,3%)
	Normal	6 (5,4%)	1 (0,9%)	5 (4,5%)
	Increased	86 (76,8%)	0 (0%)	86 (76,8%)
Leukocyte	Normal	94 (83,9%)	1 (0,9%)	93 (83,0%)
	Leukocytosis	18 (16,1%)	13 (11,6%)	5 (4,5%)
Trombocyte	Trombocytopenia	3 (2,7%)	3 (2,7%)	0 (0,0%)
	Normal	107 (95,5%)	10 (8,9%)	97 (86,6%)
	Trombocytosis	2 (1,8%)	1 (0,9%)	1 (0,9%)
NLR	Normal	97 (86,6%)	5 (4,5%)	92 (82,1%)
	Increased	15 (13,4%)	9 (8,0%)	6 (5,4%)
PT	Normal	68 (60,7%)	11 (9,8%)	57 (50,9%)
	Increased	44 (39,3%)	3 (2,7%)	41 (36,6%)
aPTT	Normal	111 (99,1%)	13 (11,6%)	98 (87,5%)
	Increased	1 (0,9%)	1 (0,9%)	0 (0,0%)
D-Dimer	Normal	36 (32,1%)	1 (0,9%)	35 (31,3%)
	Increased	76 (67,9%)	13 (11,6%)	63 (56,3%)
Procalcitonin	Normal	104 (92,9%)	6 (5,4%)	98 (87,5%)
	Increased	8 (7,1%)	8 (7,1%)	0 (0,0%)
CRP	Normal	98 (87,5%)	0 (0,0%)	98 (87,5%)
	Increased	14 (12,5%)	14 (12,5%)	0 (0,0%)
Fibrinogen	Decreased	7 (6,3%)	0 (0,0%)	7 (6,3%)
	Normal	103 (92,0%)	12 (10,7%)	91 (81,3%)
	Increased	2 (1,8%)	2 (1,8%)	0 (0,0%)

Table 3 shows fetomaternal of COVID-19. Based on Table 3, there are no infants born with positive COVID-19 status from non-COVID-19 pregnant women and COVID-19 pregnant women. There are 14 infants with negative COVID-19 status born from mother with COVID-19 (12.5%)and 98 infants born from mother without COVID-19 (87.5%).

**Table 3:** Fetomaternal Outcomes of COVID-19

Variable	Total	Mothers Condition	

		COVID-19	Non-COVID-19
COVID-19 (+)	0 (0%)	0 (0%)	0 (0%)
COVID-19 (-)	112 (100%)	14 (12,5%)	98 (87,5%)

Table 4 shows that the highest proportion of referral status among non-COVID-19 pregnant women and COVID-19 pregnant women in each group was found in the non-referral group, and the non-COVID-19 pregnant women group had the largest proportion compared to the COVID-19 pregnant women group (71.4% vs. 16.1%).

**Tabe 4:** Referral Status of Pregnant Women

Variable	Total	Mothers Condition	

		COVID-19	Non-COVID-19
Referral	22 (19,6%)	4 (3,6%)	18 (16,1%)
Non- Referral	90 (80,4%)	10 (8,9%)	80 (71,4%)

## Discussion

Pregnant women are highly susceptible to infection, including COVID-19, due to the characteristic immune response during pregnancy. This research found only 14 pregnant women with COVID-19 out of the total samples of 112 pregnant women at the USU hospital in the period 2020-2022. These results are influenced by several factors. To date, research to establish the number of comparison between pregnant women with COVID-19 and without COVID-19 is still limited. (Metz *et al.*, 2021)

COVID-19 in pregnant women can be symptomatic or asymptomatic. Most cases of COVID-19 in pregnancy will have mild symptoms, but some of the pregnant women can also experience severe symptoms especially if the pregnant woman has risk factors such as older maternal, high body mass index and comorbidities. Age is one of characteristics that is discussed in this report. Most cases of COVID-19 were found in the age group 20-35 years. This is because the distribution of pregnant women at the USU hospital during the period 2020-2022 is mostly in the 20-35 age group. Analysis Schwartz DA in 2020 of 38 pregnant women with COVID-19 in China suggested that the age range of affected women tends to be 26 to 40 years old. There was a significant association between increasing age and COVID-19 status. Severe complications of COVID-19 during pregnancy are more commonly found in symptomatic condition than non-pregnant females of reproductive age groups. Symptomatic COVID-19 in pregnancy is associated with increased risk of pregnancy complications compared with uninfected or asymptomatic pregnant females. In a systematic review by Allotey *et. al*. in 2019, it was reported that 6 – 8% of pregnant women tested positive for COVID-19, 54–77% from the cases were asymptomatic and pregnant women were more likely to be asymptomatic than non-pregnant women in the same age group (Pereira *et al.*, 2020; Rasmussen *et al.*, 2020; Nowakowski *et al.*, 2021).

Based on parity, in this research we found that most pregnant women with COVID-19 are multiparous. This finding is consistent with other studies that also found that COVID-19 pregnant women were mostly multiparous, but there is no significant association with prevalence of COVID-19 (Royal College of Obstetricians and Gynaecologists, 2020).

Based on hometown, this research found that most pregnant women with COVID-19 live in Medan. Study about the different prevalence of COVID-19 in county and village is still limited.

Laboratory findings of COVID-19 in pregnancy usually reveal abnormalities. White blood cell counts and platelets are usually lower. Meanwhile, there is an increase in CRP (C-reactive protein), LDH (lactate dehydrogenase), and PT (prolonged prothrombin time) (Satgas Penanganan COVID-19, 2020; Satuan Tugas Penanganan, 2022). This study showed that anaemia is more common in pregnant women with COVID-19. During pregnancy, women are more susceptible to anaemia because of several physiologic changes. Anaemia is considered a risk factor for severity and negative outcomes in COVID-19 patients. A meta-analysis study discovered that hemoglobin level >10 g/dL had a decreased risk of death than those with hemoglobin level <10 g/dL (Semaan *et al.*, 2022). Hemoglobin is a critical indicator of blood’s oxygen-carrying ability. As a result, low level of hemoglobin will cause decrease of oxygen transport capability to peripheral tissues. Furthermore, there is an increased demand for oxygen during COVID-19 owing to the pneumonia (Siregar and Siregar, 2021; Soheili *et al.*, 2022). Neutrophil to lymphocyte ratio is used as marker for inflammatory response that is better than single levels of neutrophils and lymphocytes count in assessing disease progression in some viral infections (Stanley *et al.*, 2020). Evidence suggests that severe COVID-19 infection is associated with increase in NLR. This parameter has been used to predict pregnancy-related complications (Terpos *et al.*, 2020). Our study showed that increased in PT is rarely found in COVID-19 group and there is only 1 patient found to have an increase in aPTT. Increased D-dimer is mostly found in non-COVID-19 patients and increase in fibrinogen is only found in 2 COVID-19 patients. Coagulopathy in COVID-19 is characterized by elevated D-dimer concentration. Elevation of D-dimer/fibrin degradation products are moreover seen in disseminated intravascular coagulation (DIC). Unlike coagulopathy associated with other underlying causes, COVID-19 is less commonly associated with prolonged PT, aPTT, or thrombocytopenia (Thachil *et al.*, 2020; Wang *et al.*, 2020). Fibrinogen appears to be well preserved, at least initially, but there are reports of low fibrinogen, especially in non-survivors (Wastnedge *et al.*, 2021). Pregnancy is physiologically hypercoagulable state. Pregnant women with COVID-19 appear to be at particularly high risk for these complications. Current RCOG advice is that all pregnant women hospitalized with confirmed or suspected COVID-19 are expected to give birth within 12 hours and are on low molecular weight heparin (LMWH) unless continued for 10 days after discharge. Prophylactic administration is recommended. (World Health Organization, 2022)

Possible vertical transmission of COVID-19 is known but appears to occur in a small number of cases of maternal coronavirus disease in late pregnancy. A number of preventive measures were recommended, including isolating the new born from the mother, refraining from breastfeeding, and washing the new born early (Wróblewska-Seniuk *et al.*, 2021). In this study, there was no infected baby who were born from mothers with COVID-19. This result is in line with the study by Wróblewska *et al (2021)* that reported no case of new born with COVID-19 who were born from infected mother (Yang *et al.*, 2020).

Referral status of pregnant women with COVID-19 in this research found 4 people were referral patients and 10 people were non referral patients. Mostly referral patients have severe conditions that are dangerous for the mother and the infants because this condition requires advanced management such as caesarean section (Liu *et al.*, 2020).

### Clinical Implications

COVID-19 is one of the deadliest public health threats. Pregnant women are a group that is very vulnerable to infection, including COVID-19. Research on the overview of COVID-19 cases in pregnancy is very important as a guide in predicting pregnant women who are susceptible to experiencing COVID-19. Data from this research can assist obstetrician in providing supervision to certain groups of pregnant women and consider management for pregnant women with COVID-19.

### Research Implications

Studies are needed to better understand how maternal and newborn outcomes have been affected by COVID-19. Our study provides a glimpse into this topic, but larger scale studies need to be done to confirm our findings. It is hoped that the results of this study can be used as a reference for further studies.

### Strengths and Limitation

The strength of this study is that samples were taken for two years so that the results of this study can describe the characteristics of COVID-19 during pregnancy more broadly.

This research has some potential limitations that should be considered. This study only involved a small number of samples so that the results of this study could not describe the actual conditions. Larger studies are needed to validate our findings. We could not distinguish between asymptomatic and symptomatic SARS-CoV-2 infection, or severity of disease, which has been shown to have different effects on pregnancy outcomes. The inclusion criteria in this study also did not include the presence of all co-morbidities of pregnant women that could affect the results of the study.

## Conclusion

Pregnant women are highly susceptible to infections, including COVID-19. Characteristic COVID-19 pregnant women at the USU Hospital in the 2021-2022 period were mostly 20-35 years old, multiparous and live in Medan. From laboratory examinations, we found out that there were alterations in CRP (C-reactive protein), LDH (lactate dehydrogenase), and PT (prolonged prothrombin time). A condition that is found in all pregnant women with COVID-19 is anaemia. From other studies reported, the vertical transmission of COVID-19 is known but appears to occur in a small number of cases of maternal coronavirus disease in late pregnancy, This finding is in line with this research where no infants with COVID-19 were found even in mother who were COVID-19 positive. In this research, we also found that fewer were the referral patients than non-referral patients. It is hoped that this research about overview of COVID-19 cases in pregnancy will increase our understanding of COVID-19 especially in pregnant women and help us develop methods to reduce the severity and spread of the disease to improve maternal and newborn outcomes.

### Competing Interests

The authors declare that there are no competing interests associated with this study.

**List of Abbreviations:**
COVID-19: (Coronavirus 2019)
SARS-CoV-2: (Severe Acute Respiratory Syndrome-Coronavirus-2)
WHO: (World Health Organization)
ACE-2: (angiotensin-converting enzyme-2)
TMPRSS2: (transmembrane biserine protease)
CRP: (C-reactive protein)
LDH: (lactate dehydrogenase)
PT: (prolonged of prothrombin time).
